# Identification of miRNA-mediated gene regulatory networks in L-methionine exposure counteracts cocaine-conditioned place preference in mice

**DOI:** 10.3389/fgene.2022.1076156

**Published:** 2023-01-19

**Authors:** Yan Wang, Lvyu Yang, Hansheng Zhou, Kunlin Zhang, Mei Zhao

**Affiliations:** ^1^ CAS Key Lab of Mental Health, Institute of Psychology, Beijing, China; ^2^ Department of psychology, University of Chinese Academy of Sciences, Beijing, China; ^3^ Department of Pharmacy, Linyi People’s Hospital, Linyi, Shandong Province, China

**Keywords:** high-throughput sequencing, microRNA, gene expression, addiction, regulatory network, L-methionine, cocaine

## Abstract

**Background and Aims:** Methionine has been proven to inhibit addictive behaviors of cocaine dependence. This study aimed to identify the potential mechanisms of MET relating to its inhibitory effects on cocaine induced cellular and behavioral changes.

**Methods:** MRNA and miRNA high-throughput sequencing of the prefrontal cortex in a mouse model of cocaine conditioned place preference (CPP) combined with L-methionine was performed. Differentially expressed miRNAs (DE-miRNAs) and differentially expressed genes (DEGs) regulated by cocaine and inhibited by L-methionine were identified. DEGs were mapped to STRING database to construct a protein-protein interaction (PPI) network. Then, the identified DEGs were subjected to the DAVID webserver for functional annotation. Finally, miRNA-mRNA regulatory network and miRNA-mRNA-TF regulatory networks were established to screen key DE-miRNAs and coregulation network in Cytoscape.

**Results:** Sequencing data analysis showed that L-methionine reversely regulated genes and miRNAs affected by cocaine. Pathways associated with drug addiction only enriched in CS-down with MC-up genes targeted by DE-miRNAs including GABAergic synapse, Glutamatergic synapse, Circadian entrainment, Axon guidance and Calcium signaling pathway. Drug addiction associated network was formed of 22 DEGs including calcium channel (Cacna1c, Cacna1e, Cacna1g and Cacng8), ephrin receptor genes (Ephb6 and Epha8) and ryanodine receptor genes (Ryr1 and Ryr2). Calcium channel gene network were identified as a core gene network modulated by L-methionine in response to cocaine dependence. Moreover, it was predicted that Grin1 and Fosb presented in TF-miRNA-mRNA coregulation network with a high degree of interaction as hub genes and interacted calcium channels.

**Conclusion:** These identified key genes, miRNA and coregulation network demonstrated the efficacy of L-methionine in counteracting the effects of cocaine CPP. To a certain degree, it may provide some hints to better understand the underlying mechanism on L-methionine in response to cocaine abuse.

## 1 Introduction

Repeated drug abuse causes multifaceted alterations to neural circuits through changes in gene expression and DNA methylation. Stable changes in gene expression caused by addictive drugs are believed to induce neuronal adaptations in synaptic plasticity (e.g., long-term potentiation and depression) (Nestler, 2002). Research over the past decade has also identified several molecular pathways of learning and memory that are strongly involved in drug addiction (Nestler, 2002). However, how the activities of these various signaling cascades are coordinated to regulate cocaine addiction remains unknown.

MicroRNAs, small, non-coding RNAs highly abundant in the brain (Most et al., 2014), control mRNA translation in the cell and spatially restricted sites such as the synapses and can mediate activity in various intracellular signaling cascades (Hollander et al., 2010; Most et al., 2014). Recent studies have demonstrated the crucial role of miRNAs in the modulation of cocaine addiction in both rodent models and humans. The expression of miR-181a, let-7d, and the brain-specific miR-124 are all induced by exposure to cocaine in the nucleus accumbent (Chandrasekar and Dreyer, 2009). The overexpression of let-7 could potentially attenuate cocaine-induced CPP (Chandrasekar and Dreyer, 2011), while elevated expression of mature miR-212 and miR-132 has persistent effects during cocaine-free periods following prolonged drug consumption. MiR-212 signaling plays a key role in determining vulnerability to cocaine addiction (Hollander et al., 2010; Sadakierska-Chudy et al., 2017). In addition, miR-124 and miR-181 expression is upregulated in the blood of cocaine users (Viola et al., 2019).

L-Methionine (MET) is an essential sulfur-containing amino acid involved in protein synthesis, including the synthesis of various neurotransmitters in the brain that play critical roles in proper nerve growth, development, and function (Vuaden et al., 2012; Ringman and Coppola, 2013). Long-term methionine exposure reportedly causes locomotor retardation, memory impairment, and anxiety (Viggiano et al., 2012; Vuaden et al., 2012), while restriction of dietary methionine is associated with increased longevity and decreased incidence of age-related disorders in mice and rats (Zimmerman et al., 2003; Miller et al., 2005; Malloy et al., 2006). More importantly, our initial findings and those of others suggest that methionine administration can inhibit addictive behaviors in rodent models of cocaine dependence (Tian et al., 2012; Wright et al., 2015; Tian et al., 2016).

Methionine metabolism occurs through the transmethylation and trans-sulphuration pathways. S-adenosylmethionine (SAM), an important methyl donor (Bolander-Gouaille and Bottiglieri, 2007; Sanderson et al., 2019), is generated *via* the transmethylation pathway through the activity of the methionine adenosyltransferase enzyme and is predicted to affect DNA methylation reactions in the whole genome. Alterations in DNA patterns in the brain can produce long-lasting changes in gene expression, which affect behavior (Tsankova et al., 2007; Maze and Nestler, 2011). In contrast, SAM increased locomotor sensitization in mice *via* the modification of cocaine-induced DNA methylation (Anier et al., 2013), suggesting that MET is functionally distinct from SAM. However, the specific mechanisms of how methionine affects drug-induced behaviors remain unclear.

Based on this evidence, this study aimed to identify the potential mechanisms of MET relating to its inhibitory effects on cocaine-induced cellular and behavioral changes. We examined the effects of L-methionine on cocaine-induced conditioned place preference and used a combination of high-throughput transcriptome sequencing and miRNA sequencing to identify target genes regulated by L-methionine in the PFC upon cocaine treatment.

## 2 Materials and methods

### 2.1 Animal hosts and drug treatments

Animal hosts and drug treatments were performed as described previously (Wright et al., 2015). Briefly, adult male C57/BL6 mice (20–30 g) were housed under a 12 h light/dark cycle supplemented with food and water *ad libitum*. All animal experiments were approved by the Institute Ethics Committee of the Institute of Psychology, CAS.

During the methionine (Sigma, United States) experiments, the mice were injected subcutaneously with 1 g/kg (6.6 mmol/kg) L-methionine twice daily for 10 consecutive days using the CPP procedure (including pre-test, training, and post-test days). During training, the mice were injected with methionine 1 h before each behavioral experiment. The animals were divided into four groups: 1) saline + saline (SS), 2) MET + saline (MS), 3) MET + cocaine (MC), and 4) cocaine + saline (CS).

### 2.2 Conditioned place preference

An unbiased conditioning protocol was used as described previously (Walters and Blendy, 2001). In brief, the mice were placed into a middle chamber and allowed to roam freely between the two side chambers for 15 min on day 1, and then arranged into control and experimental groups with equivalent pre-test scores. After experimental manipulation, the mice were paired up for 8 days. The saline group received saline in both sides of the chambers, while the drug groups were injected with cocaine (20 mg/kg, i.p., Qinghai Pharmaceutical Group Co. LTD., China) and saline on one side and saline only on the opposite side (Romieu et al., 2008). After each manipulation, the mice were confined to the corresponding conditioning chambers for 30 min before being returned to their cages. On the test day, the mice were allowed access to all chambers and a place preference score (CPP score) was assigned by subtracting the time spent in the drug-paired chamber from the time spent in the saline-paired chamber.

### 2.3 RNA extraction

Two hours after the (day 10) CPP test, the animals were sacrificed and their mPFCs were surgically excised. The tissue was stored in liquid nitrogen immediately after extraction and then transferred to −80°C. Total RNA was extracted from the frozen tissues using the TIANamp DNA/RNA Isolation Kit (TIANGEN), including additional treatment with RNase-free DNase I (Ambion) for 30 min at 37°C to remove contaminating DNA.

### 2.4 RNA sequencing and bioinformatics

A total of 1 µg of RNA was taken from a pool of two animals (500 ng each) with three biological replicates per experimental condition to generate an mRNA sequencing library as follows: briefly, after poly-A addition to the mRNA molecules, the mRNA was fragmented. The cleaved RNA fragments were then copied into first-strand cDNA before second-strand cDNA synthesis. A single ‘A’ base was added to the cDNA, to which an adaptor was ligated and enriched by PCR amplification. Single-strand DNA circles (ssDNA circles) were constructed and DNA nanoballs (DNBs) were generated. These DNBs were loaded into the patterned nanoarrays and pair-end reads of 100 bp were read on the BGISEQ-500 platform at Beijing Genomics Institute (BGI; Shenzhen, China).

Raw sequencing reads were filtered for clean reads using SOAPnuke (Mak et al., 2017; Chen et al., 2018) (v1.5.2, parameters −l 15, −q 0.2, −n 0.05) (https://github.com/BGI-flexlab/SOAPnuke). The HISAT pipeline (Kim et al., 2015) was applied to the align reads to the reference genome (mm10). StringTie (Pertea et al., 2015) was then used for transcriptional reconstruction. Subsequently, Cuffcompare (Trapnell et al., 2012) (Cufflinks tools) was utilized to compare the reconstructed transcripts and estimate the expression levels (FPKM, Fragments Per Kilobase of exon model per Million mapped fragments) of all detected isoforms. The coding potentials of the novel transcripts were predicted by CPC (Kong et al., 2007). The identification of DEGs (differentially expressed genes) was based on the negative binomial distribution of the DEseq2 package (Love et al., 2014). The cutoff of DEGs was *p*< 0.1.

### 2.5 MiRNA sequencing and bioinformatics

A total of 1 µg of RNA from a pool of two animals (500 ng each) with three biological repeats per experimental condition was used to generate a miRNA sequencing library. RNA segments 18–30 nt in length were separated and recovered by PAGE. The subsequent steps were the same as those used to generate the mRNA sequencing library from the poly-A addition. The miRNA DNBs (DNA nanoball) were loaded into the patterned nanoarrays and 50-bp single-end reads were read through on the BGISEQ-500 platform at Beijing Genomics Institute (BGI; Shenzhen, China).

The raw sequencing reads were filtered with FASTQ to remove reads with low quality, adapter contamination, and lengths <16 nt (Cock et al., 2010). The clean reads were mapped to the reference genome using AASRA (Tang et al., 2017). Reads matching rRNAs and tRNAs were excluded. The remaining reads were aligned against the miRBase (v21) (Griffiths-Jones et al., 2008) using Bowtie allowing one mismatch. Unaligned sequences were pooled to identify novel miRNAs using miRDeep (v2.0.0.5) (An et al., 2013) with the default parameters. To investigate the expression profiles of miRNAs, the frequency of miRNA counts was normalized to TPM (tags per million) using the following formula: normalized expression = actual miRNA read count/total clean read count × 106.

Differentially expressed miRNAs between the paired groups were analyzed using DEGseq (Wang et al., 2010). The *p*-values calculated for each gene were adjusted to Q-values for multiple testing corrections using two alternative strategies (Hochberg, 1995; Storey and Tibshirani, 2003). To improve the overall accuracy of the DEGs results, a gene was defined as a DE-miRNA (differentially expressed miRNA) when the Q-values was ≤ 0.05 and the |log2fold_change| was >0.5.

### 2.6 MiRNA target prediction

The potential miRNA targets were identified using miRanda (v3.3a, parameters -en -20 -strict) (Enright et al., 2003; Betel et al., 2008) and TargetScan (v6.0, parameters-c 4) (Agarwal et al., 2015). Sequences predicted by both miRanda and TargetScan were considered miRNA targets. Furthermore, if there are more than 100 target genes predicted for a miRNA, the top 100 target genes were selected according to the scoring system. To reduce the false identification rate, the gene expression profiles of putative targets were required to be negatively correlated with miRNA profiles.

### 2.7 Establishment of PPI networks and module analysis

A STRING web-based tool database (https://string-db.org) was used to construct the PPI network of DEGs affected by cocaine and reversed by MET. The PPI network was constructed by mapping the genes to the STRING database with confidence scores of 0.4. Cytoscape (v3.9.0) was used to visualize and analyze the PPI networks (Shannon et al., 2003).

To identify intersecting clusters in the PPI networks, we used the Molecular Complex Detection plugin (MCODE) Cytoscape plugin with identification criteria including a degree cutoff of 2, a node score cutoff of 0.2, a k-core of 2, and a maximum depth of 100. Significant modules were those with MCODE scores of ≥4 and nodes ≥6, which constrained the cluster size for co-expressing networks (Bader and Hogue, 2003).

### 2.8 Construction of a TF-miRNA-mRNA network

The Transcriptional Regulatory Relationships Unraveled by Sentence-based Text mining (TRRUST, http://www.grnpedia.org/trrust/) database (Han et al., 2018) was used to predict TFs regulating the DEGs based on existing literature. Subsequently, TransmiR 2.0 (Tong et al., 2019) was used to predict the potential target transcription factors of miRNA with validated information. Finally, a TF-miRNA-mRNA coregulation network was constructed to show the potential molecular mechanisms of cocaine initiation and extinction. Cytoscape was used to visualize the interactions in the TF-miRNA-mRNA coregulation network.

### 2.9 Gene enrichment analysis

Functional annotation of GO and KEGG pathway enrichment was performed using the web-based DAVID v6.8 tool (Huang da et al., 2009). Gene and miRNA expression regulation and KEGG visualized with heatmap, volcano, bar plot, and bubble plot were plotted by https://www.bioinformatics.com.cn, an online platform for data analysis and visualization.

### 2.10 Real-time PCR validation

DEGs were validated by quantitative reverse-transcription PCR (RT-qPCR) using the Maxima SYBR Green qPCR Master Mix kit (Fermentas) according to the manufacturer’s instructions on an ABI Prism 7500 Sequence Detection System machine (Applied Biosystems Inc.). All real-time RT-qPCR data were normalized to SS expression level (see the additional data file for primer information).

### 2.11 Statistical analysis

Data are expressed as means ± SEM. Statistical analysis of qPCR data was performed using an unpaired *t* test with two tailed distributions using GraphPad Prism version 6.0. The results were considered statistically significant when *p* < 0.05.

## 3 Results

### 3.1 Effects of methionine on cocaine-induced behaviors

We previously showed that L-methionine inhibits cocaine-induced behaviors (Zhang et al., 2021). Briefly, comparisons before and after drug treatment (pre-test and pro-test) for each group showed significant differences only in the cocaine-induced CPP group (*p* < 0.001)(Supplementary Figure S1). *t*-Test analysis of the pre-test data revealed that methionine administration significantly attenuated cocaine-CPP expression (CS vs. MC *p* < 0.05).

### 3.2 GO and KEGG pathways induced by cocaine

To determine the gene expression changes in the whole genome underlying the effects of cocaine CPP and methionine in response to cocaine CPP, we first performed mRNA-sequencing of the extracted PFCs of mice from each of the four treatment groups (SS: saline+saline, CS: cocaine+saline, MS: MET+saline, and MC: MET+cocaine). A total of approximately 50 million 100-bp, paired-end reads were obtained per sample after removing low-quality reads. Approximately 90% of the clean reads mapped to the genome and 55% of the clean reads uniquely mapped to the genome (Supplementary Table S2).

**FIGURE 1:**
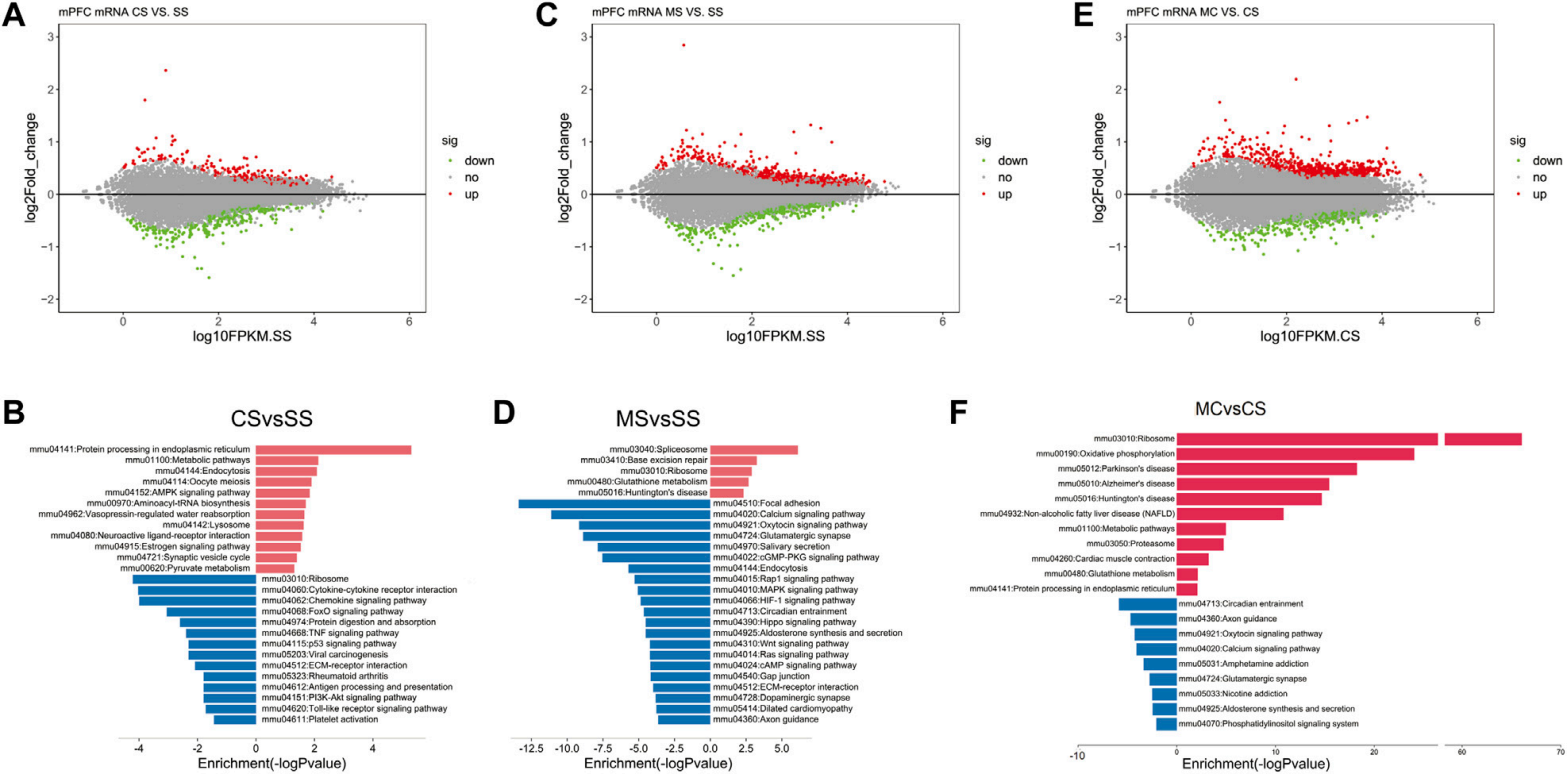
Cocaine and L-methionine (MET) cause broad changes to the transcriptome. mRNA from the prefrontal cortexes (PFCs) of SS (saline + saline), CS (saline + cocaine), MS (MET + saline), and MC (MET + cocaine) mice were subjected to RNA sequencing and analysis as described in the methods. **(A)** MA-plot [M (log ratios); A (mean average)] of differentially expressed genes (DEGs) in the CS group (*p* < 0.1). The log2 fold change values for CS vs. SS are plotted against the average log expression values (fragments per kilobase of transcript per million mapped reads [FPKM]). **(B)** Enriched pathways of significantly down and upregulated genes in the CS group (*p* < 0.05). **(C)** MA-plot of differentially expressed genes (DEGs) in the MS group (*p* < 0.1). Log2 fold change values for MS vs. SS are plotted against the average log expression values (FPKM). **(D)** Enriched pathways of significantly down and upregulated genes in the MS group (*p* < 0.05). **(E)** MA-plot of DEGs in the MC group (*p* < 0.1). Log2 fold change values for MC vs. CS are plotted against the average log expression values (FPKM). **(F)** Enriched pathways of significantly down and upregulated genes in the MC group (*p* < 0.05). In the MA plot, upregulated and downregulated DEGs are shown in red and green, respectively.

Analysis of the mRNA sequencing data in the CS group compared to the SS group revealed 655 DEGs (Figure 1A), including 333 upregulated genes and 322 downregulated genes. KEGG pathway analysis of the upregulated and downregulated DEGs identified certain pathways enriched in the upregulated genes that were functionally associated with drug addiction, including mmu04721: synaptic vesicle cycle (Luscher and Ungless, 2006) and mmu04080: neuroactive ligand-receptor interaction and AMPK signaling pathway (Gao et al., 2017) (Figure 1B). Moreover, the results of the GO analysis in the upregulated genes showed enrichment for GO terms that directly corresponded with the characteristics of cocaine addiction, including response to cocaine, locomotor behavior, and regulation of synaptic plasticity (Supplementary Table S3).

### 3.3 mRNA-seq revealed that L-methionine downregulates addiction pathways

As shown in Supplementary Figure S1, MET did not alter behavior in the saline-treated group. To determine how MET treatment modified the mouse transcriptome, we simultaneously performed mRNA sequencing of the MS group. A total of 1531 DEGs were identified in the MS group compared to the SS group, including 204 upregulated DEGs and 674 downregulated DEGs (Figure 1C). KEGG pathway analysis of the upregulated and downregulated DEGs revealed that the addiction-related pathways; for example, the calcium signaling pathway, glutamatergic synapse, the MAPK signaling pathway, the cAMP signaling pathway, and dopaminergic synapse, were only enriched in the presence of the downregulated genes (Figure 1D). As the MET treatment did not induce CPP, we speculated that the downregulated genes enriched in drug addiction-related pathways did not induce CPP.

### 3.4 KEGG analyses revealed that L-methionine downregulates addiction pathways following cocaine CPP

Next, we applied the same analyses to study the effect of L-methionine on cocaine CPP. We first identified induced by repeated cocaine treatment and inhibited by MET treatment. We performed a differential expression analysis between the MC and CS groups to obtain a list of DEGs affected by MET treatment in the context of cocaine treatment. A total of 644 DEGs were upregulated and 352 DEGs were downregulated in the MC-treated mice compared to the CS-treated mice (Figure 1E). The KEGG analysis plot showed that most of the pathways enriched with downregulated genes were associated with drug addiction, including those related to amphetamine addiction, glutamatergic synapse, nicotine addiction, GABAergic synapse, cocaine addiction, morphine addiction, etc. (Figure 1F). Combine with the results observed for MET treatment only, these findings strongly indicated that MET inhibited cocaine CPP by downregulating these addiction-related pathways.

### 3.5 L-methionine inhibits DEGs induced by cocaine

**FIGURE 2:**
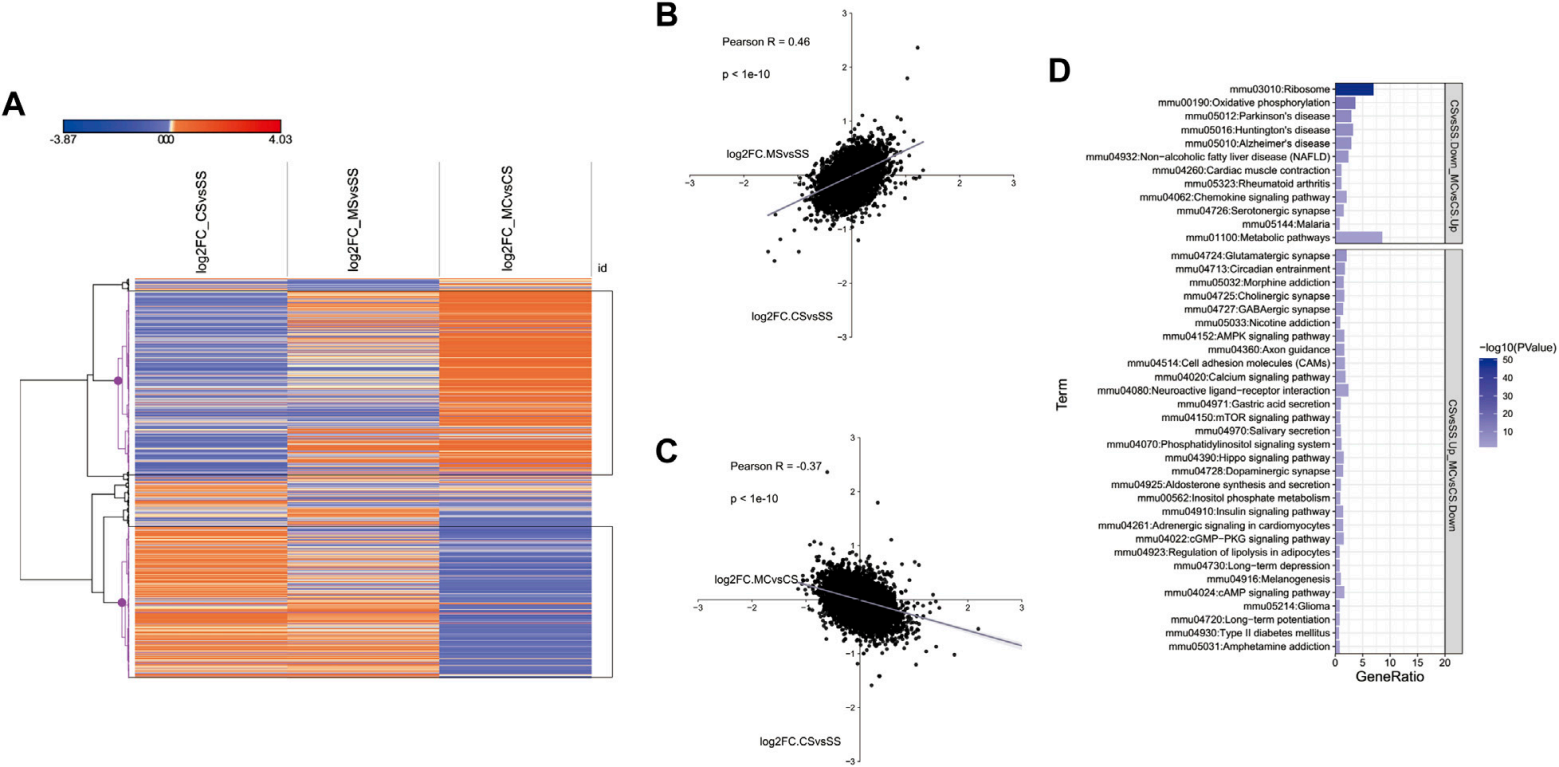
Transcriptome-wide response of L-methionine (MET) to cocaine CPP. **(A)** Heatmap of all significantly differentially expressed genes in each group (saline + cocaine [CS], MET + saline [MS], and MET + cocaine [MC]). Red, upregulated; blue, downregulated. **(B,C)** Scatter plot of DEGs depicting the expression changes between MS and CS **(B)** and between MC and CS **(C)**. **(D)** Enriched pathways of DEGs downregulated in the MC group and upregulated in the CS group, and upregulated in the MC group and downregulated in the CS group.

We showed above that the MET treatment downregulated addiction-related pathways (Figures 1D, F). To further investigate the genes induced by cocaine and inhibited by MET, we plotted the log2fold_change of DEGs in the CS, MS, and MC groups in a heatmap. The resulting hierarchical clustering indicated that many genes exhibited fold changes in opposite directions in the MC mice compared to those in the CS and MS mice (Figures 2A–C). These genes were clustered into two groups as marked in black: genes inhibited by cocaine and induced by L-methionine (CS-down and MC-up) and the reverse: genes induced by cocaine and inhibited by L-methionine (CS-down and MC-up). The KEGG pathways of these two groups were analyzed separately to show that the pathways of the CS-down and MC-up genes were enriched for neurodegenerative diseases while the CS-up and MC-down genes were enriched for substance dependence (morphine addiction and nicotine addiction) and nervous function (glutamatergic synapse, cholinergic synapse, and GABAergic synapse) pathways (Figure 2D).

**FIGURE 3:**
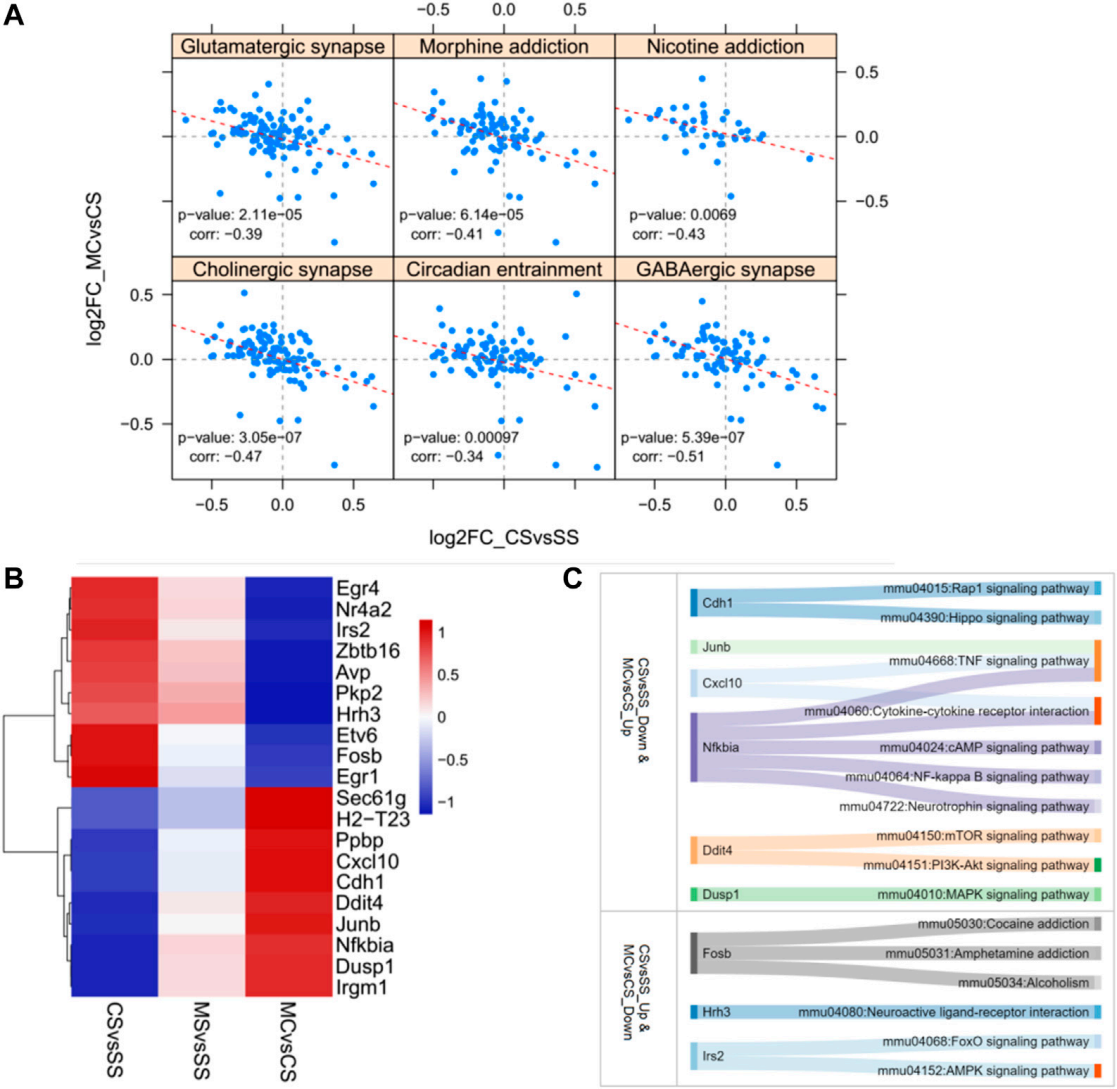
Reversely regulated genes by L-methionine (MET) response to cocaine CPP. **(A)** Scatter plot of all gene expression levels in substance dependence and drug abuse-related pathways. **(B)** Heatmap of the top 20 DEGs regulated by cocaine and reversed by MET. **(C)** Sankey diagram depicting the involved pathways of the top 20 DEGs.

Posthoc analysis of the log2fold changes in genes related to substance dependence and nervous function in a less stringent manner showed a significant negative relationship between the CS and MC groups (*p* = 0.0000614 for morphine addiction, 0.0069 for nicotine addiction, 0.0000211 for glutamatergic synapse, 0.000000539 for GABAergic synapse, 0.000000305 for cholinergic synapse, and 0.00097 for circadian entrainment) (Figure 3A). This further supported the role of MET in reversing the addiction pathways modified by cocaine treatment by reversing the activation of genes in these pathways. We also plotted the top 10 most significant genes in the CS-down and MC-up group and the CS-down with MC-up group to identify key genes involved in drug addiction (Figures 3B, C). Among them, Fosb, Hrh3, Ins2, and Nfkbia were involved in drug addiction-associated pathways.

### 3.6 PPI network construction and module identification

**FIGURE 4:**
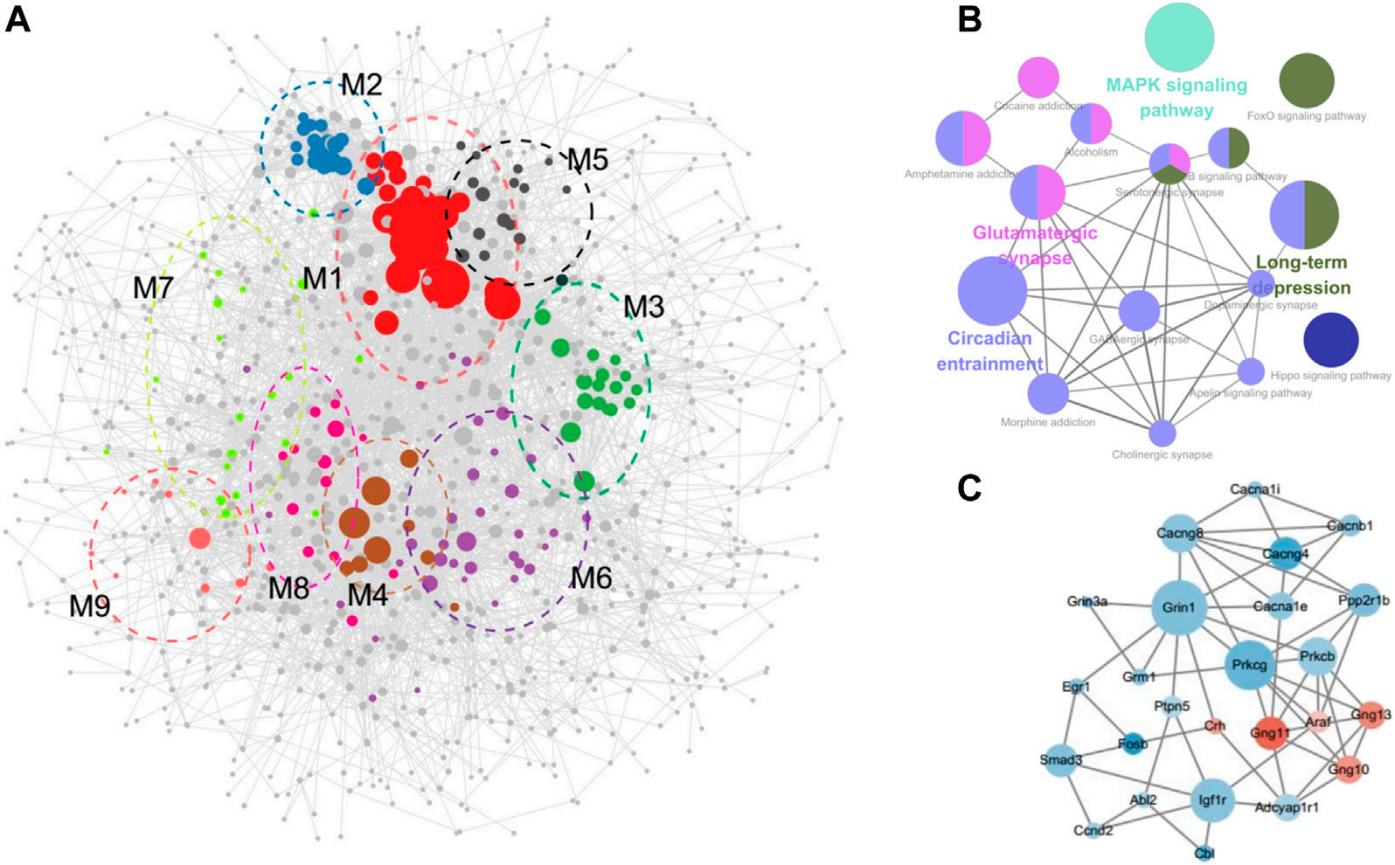
PPI network construction. **(A)** PPI network with modules comprising the DEGs regulated by cocaine and reversed by MET. **(B)** Visualization of the enrichment of KEGG terms associated with addiction using the ClueGO/CluePedia plugin from Cytoscape. **(C)** Subnetwork constructed with genes from modules 6–9 enriched in pathways associated with drug reward. Green, downregulated; red, upregulated.

STRING provides original reliable protein data for consequent analysis. DEGs induced by cocaine and reversely regulated by L-methionine were mapped to generate PPI networks containing 1,040 nodes and 5,946 edges (Figure 4A). The top 10 hub genes of the total DEGs PPI network were Rps27a, Hras, Cdh1, Mapt, Acta2, Grin1, Isg15, Smad3, Nfkbia, and Trrap (Supplementary Table S4).

The overall PPI network of the DEGs was surveyed to identify functional modules in the network (Figure 4A; Table 1; Supplementary Table S5). Nine modules were identified in the PPI network, with MCODE scores ≥4 and nodes ≥6. To further investigate the functions of these modules, we performed pathway and GO analysis. In some modules, most genes were enriched in the same pathway categories. For example, most genes were enriched in ribosomes in module M1, while module M5 was enriched for genes related to the spliceosome. Meanwhile, module M4 was enriched for pathways associated with neurodegenerative diseases such as Parkinson’s disease, Alzheimer’s disease, and Huntington’s disease.

**TABLE 1:** GO and KEGG pathways enriched in each module.

Mode	Score	No. Node	KEGG_Pathway/GO:BP	Gene ratio (%)	*p*-value
M1	55.05	65	mmu03010:Ribosome	76.92	4.30E-69
			mmu03060:Protein export	6.15	0.015614
M2	16.00	18	mmu05012:Parkinson’s disease	83.335	8.33E-24
			mmu05010:Alzheimer’s disease	83.335	1.02E-22
			mmu05016:Huntington’s disease	83.335	5.20E-22
M3	11.50	17	mmu04623:Cytosolic DNA-sensing pathway	17.65	6.71E-04
			mmu04622:RIG-I-like receptor signaling pathway	17.65	7.57E-04
			mmu05160:Hepatitis C	17.65	0.002997
M4	7.75	9	mmu04514:Cell adhesion molecules (CAMs)	33.33	0.0062
			mmu05216:Thyroid cancer	22.22	0.022
M5	7.50	15	mmu03040:Spliceosome	31.25	4.22E-07
M6	5.15	27	GO:0006366∼transcription from RNA polymerase II promoter	25.93	0.000029
			GO:0007411∼axon guidance	25.93	0.00030
			GO:0051591∼response to cAMP	14.81	0.00046
M7	5.08	20	mmu04010:MAPK signaling pathway	25	4.46E-04
			mmu00562:Inositol phosphate metabolism	20	5.50E-04
			mmu04260:Cardiac muscle contraction	15	0.006119
M8	4.85	11	mmu04727:GABAergic synapse	27.78	7.06E-06
			mmu05032:Morphine addiction	27.78	9.21E-06
			mmu04713:Circadian entrainment	27.78	1.13E-05
M9	4.15	9	mmu04080:Neuroactive ligand-receptor interaction	33.33	0.00097
			mmu04020:Calcium signaling pathway	22.22	0.0074

The pathways in module M8 were involved in substance dependence and nervous organismal systems. Morphine addiction, GABAergic synapse, and circadian entrainment were represented in this module. Module M7 was enriched in the cardiac muscle contraction and MAPK signaling pathways, which was a signal transduction pathway and reportedly involved in drug addiction and possibly required for the establishment of NAc amphetamine-produced CPP (Gerdjikov et al., 2004; El Rawas et al., 2020). Moreover, module M9 was enriched in the circadian rhythm pathway, which is reported as the common pathway underlying drug abuse (Li et al., 2008). However, though no KEGG pathways were assigned to Module M6, the GO terms for the biological processes of response to cAMP were enriched in this module. cAMP is a second messenger involved in the molecular network of drug addiction (Li et al., 2008).

The GO and KEGG analysis of the modules showed that the functions of modules M6–M9 were involved in drug addiction. Pathway networks were visualized using the ClueGO plugin (Figure 4B). Additionally, subnetworks of the DEGs enriched in the drug addiction-related pathways from modules M6–M9 were generated (Figure 4C). The subnetworks mainly comprised calcium channel (cacna1e, cacna1i, cacnb1, cacng4, and cacng8), glutamate receptor (grin3a, grin1, and grm1), G protein (gng10, gng11, and gng13), protein kinase C (prkcb and prkcg), and immediate early (Egr1 and Fosb) genes.

### 3.7 Effects of MET on miRNA expression

MicroRNAs (miRNAs) are key mediators of the silencing of post-transcriptional gene expression. We performed small RNA-seq to reveal the miRNome of the MET response to cocaine addiction. The miRNAs were to obtain approximately 12 million reads for each sample, of which ∼15% of reads were filtered out for low quality or short length (<15 nt) (Supplementary Table S6).

**FIGURE 5:**
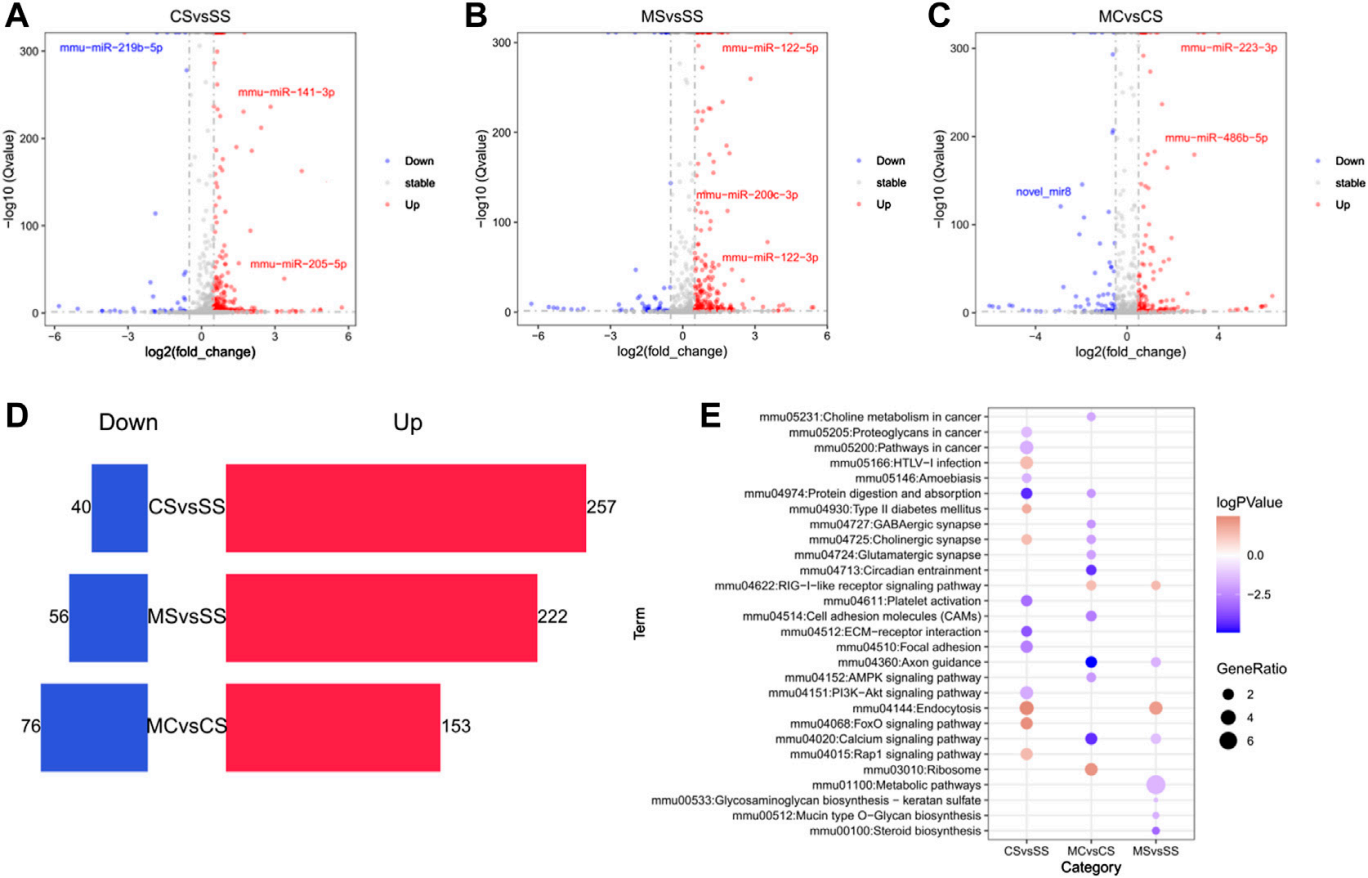
Global alteration of miRNA in cocaine addiction and MET treatment response to cocaine addiction. **(A–C)** Volcano plot of differentially miRNAs (DE-miRNAs) in the CS **(A)**, MS **(B)**, and MC **(C)** groups (*q* < 0.05 and |log2fold_change>=0.5|). Blue, downregulated miRNAs; red, upregulated miRNAs. **(D)** Bar chart of the number of DE-miRNAs in the CS, MS, and MC groups. **(E)** Bubble diagram of enriched pathways of the predicted target DEGs of DE-miRNAs in the CS, MS, and MC groups (*q* < 0.05).

Compared to the SS group, 297 differentially expressed miRNAs (DE-miRNAs) were identified using DEseq2 in the cocaine-treated group (CS). Among these, 257 miRNAs were significantly highly induced by cocaine treatment, while 40 miRNAs showed significantly low expression (Figures 5A, D; Supplementary Table S7). To predict their target genes, we filtered the DEGs mediated by differentially expressed miRNA (DEGs-miRNA) according to the miRNA target prediction rules. Our findings showed enrichment of the cholinergic synapse and FoxO signaling pathways with upregulated DEGs-miRNA (Figure 5E). Meanwhile, compared to the SS group, the MS group showed 222 significantly high expressed miRNAs and 56 significantly lowly expressed miRNAs following CPP (Figures 5B, D; Supplementary Table S7). KEGG pathway analysis of miRNA targeting prediction showed that the axon guidance and calcium signaling pathway were enriched in downregulated predicted DEGs (Figure 5E).

The same analysis was applied to the MC group. We compared the MC group to the CS group to assess the effect of MET on response to cocaine. We identified 153 upregulated miRNAs and 76 downregulated miRNAs (Figures 5C–E). KEGG pathway analysis showed that most pathways enriched in downregulated-predicted DEGs were associated with drug addiction, including the GABAergic synapse, cholinergic synapse, glutamatergic synapse, circadian entrainment, axon guidance, and calcium signaling pathways, consistent with the pathways enriched by DEGs and indicating that MET acted on miRNAs to regulate gene expression.

### 3.8 MET acts on miRNAs to downregulate addiction-related pathways following cocaine CPP

**FIGURE 6:**
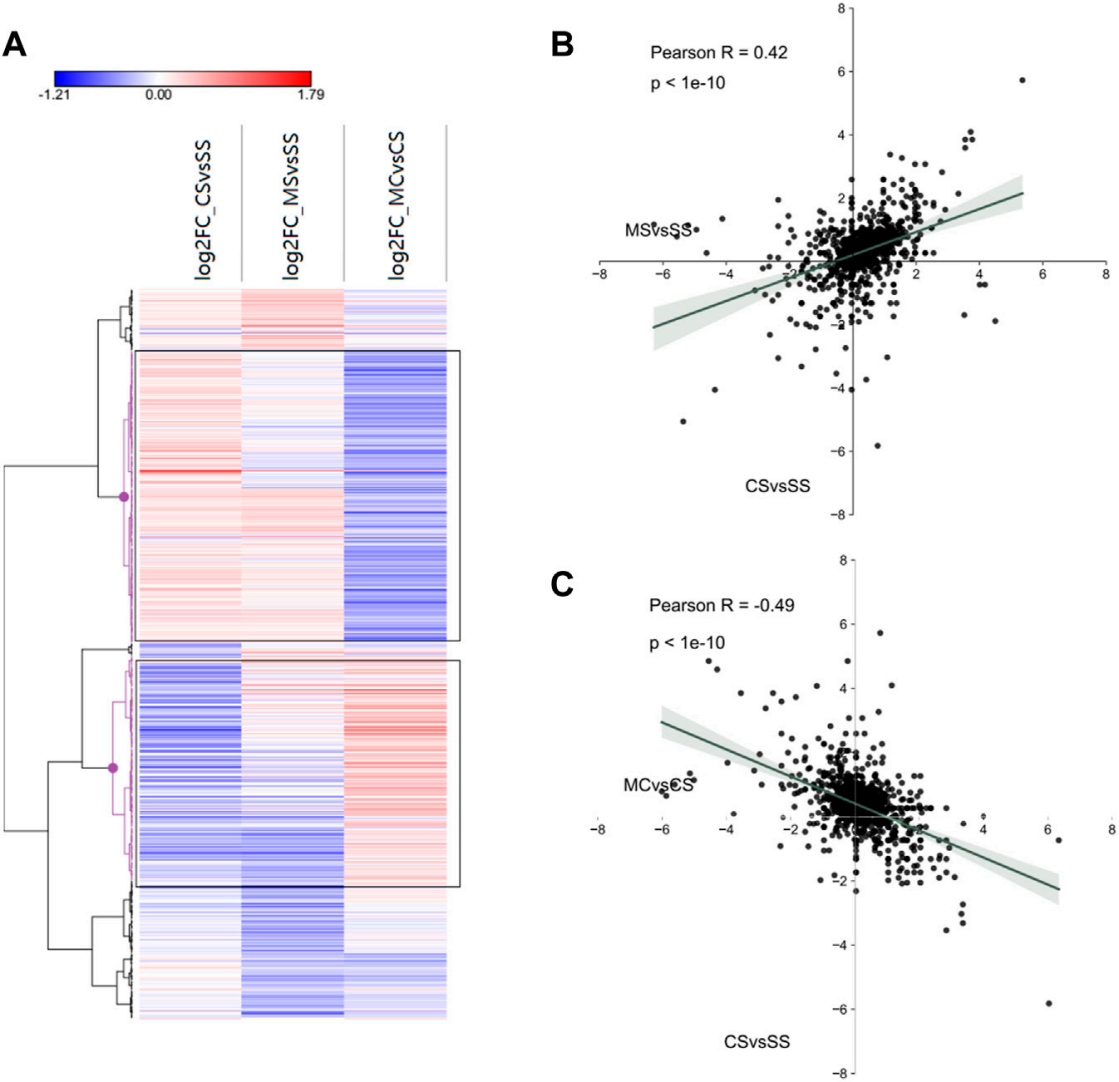
Interactions between miRNA and mRNA of cocaine addiction and MET treatment response to cocaine addiction. **(A)** Heat map of all significant miRNAs in each group (saline + cocaine [CS], MET + saline [MS], and MET + cocaine [MC]). Red, upregulated; blue, downregulated. **(B,C)** Scatter plot of DE-miRNAs depicting expression changes between the MS and CS **(B)** and between the MC and CS **(C)** groups.

To further investigate miRNA regulation in the MET treatment of cocaine CPP, we first plotted the log2fold_changes of DE-miRNAs in the CS, MS, and MC conditions as a heat map. As expected, a significant number of miRNAs exhibited fold changes in opposite directions in the MC mice compared to the CS mice, which was confirmed by hierarchical clustering marked in black (Figure 6A). We then plotted the fold-changes between the CS and MC groups and between the CS and MS groups, which showed a positive relationship (R = 0.42) between CS and MS and a negative relationship (R = −0.49) between CS and MC, consistent with the relationship of DEGs, indicating that MET inhibited cocaine CPP behavior by reversing the DEGs and DE-miRNAs regulated by cocaine (Figures 6B,C).

**FIGURE 7:**
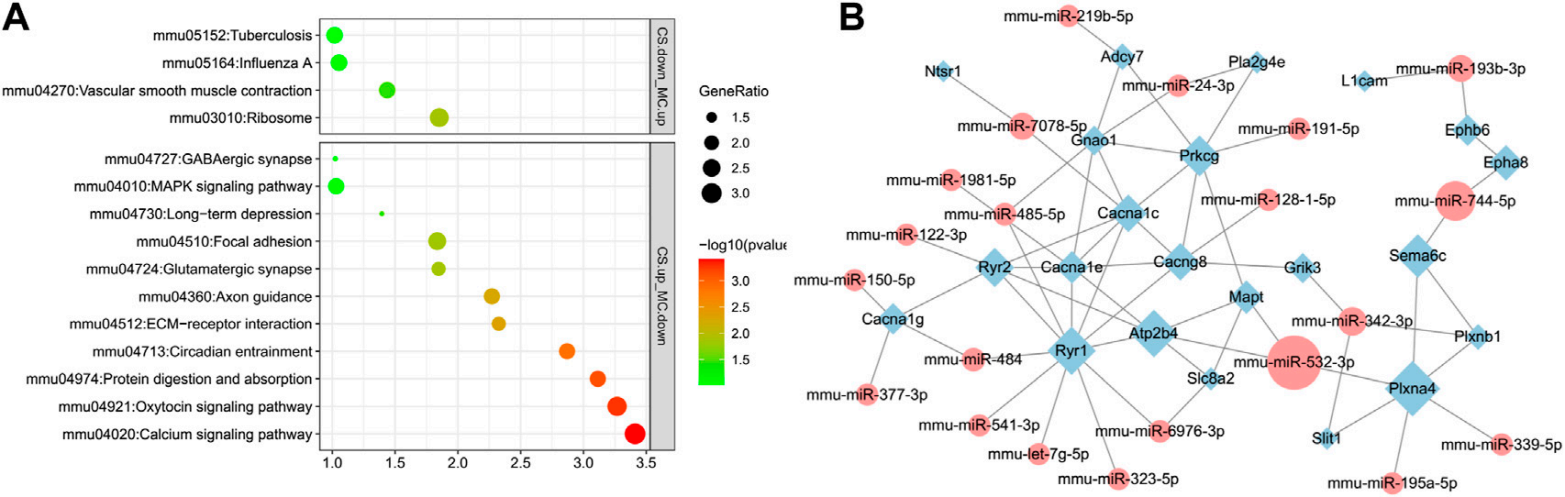
Pathways and network visualization of the DEGs targeted by DE-miRNAs induced by cocaine and inhibited by MET. **(A)** Enriched pathways of predicted DEGs targeted by DE-miRNAs downregulated in the MC group and upregulated in the CS group, and upregulated in the MC group and downregulated in the CS group. **(B)** Network of DE-miRNAs and their target DEGs in the enriched substance dependence and addiction-related pathways. Diamonds, DEGs; ellipses, miRNAs; green, downregulated; red, upregulated.

KEGG pathway analyses of DEG-miRNAs (DEGs regulated by DE-miRNAs) in the CS upregulation with MC downregulation subgroup and CS downregulation with MC upregulation subgroup were performed separately. The pathways associated with drug addiction were only enriched in the CS downregulation with MC upregulation subgroup, including the GABAergic synapse, glutamatergic synapse, circadian entrainment, axon guidance, and calcium signaling pathways (Figure 7A). These pathways were top-ranked pathways enriched with DEGs induced by cocaine and downregulated by L-methionine, indicating that the expression of these genes located in these pathways might be regulated by miRNAs in responses to cocaine and MET.

We then selected genes in the substance dependence, synapse, and other addiction-related pathways in addition to their source miRNAs to create a network. A total of 32 miRNAs targeted 22 genes, including those related to calcium channels (Cacna1c, Cacna1e, Cacna1g, and Cacng8), ephrin receptors (Ephb6 and Epha8), and ryanodine receptors (Ryr1 and Ryr2). Calcium channel genes were also present in the addiction-associated DEG PPI network shown in Figure 4C, indicating that the calcium channel gene network is a core gene network that plays an important role in the process of L-methionine counteracting cocaine CPP (Figure 7B).

### 3.9 TF-miRNA-mRNA network

**FIGURE 8:**
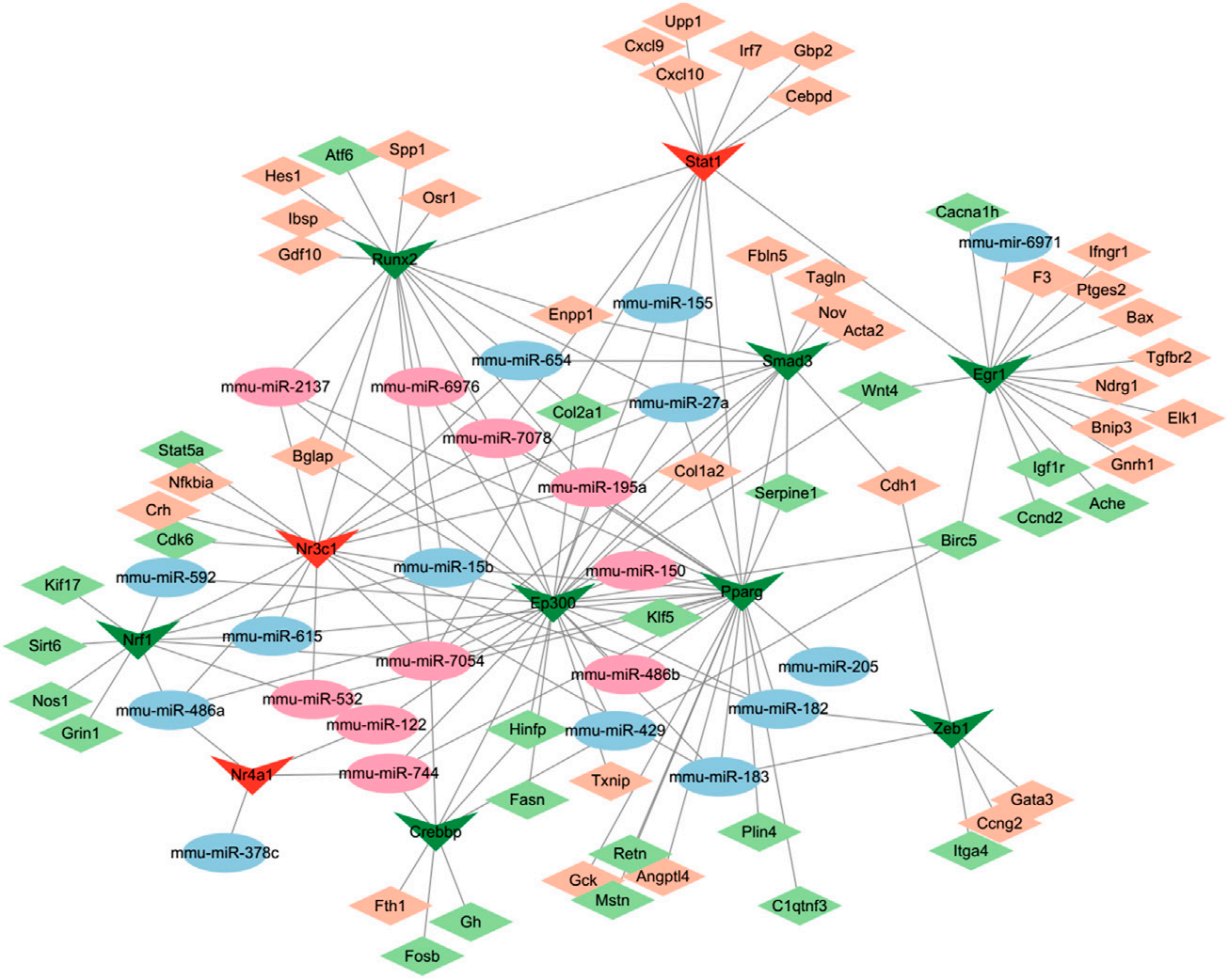
Gene regulatory network incorporating DEGs–TFs–miRNAs regulated by cocaine and reversed by MET. Diamonds, DEGs; Vs, TFs; ellipses, miRNAs. Green and blue, downregulated; red and darksalmon, upregulated.

MiRNAs and transcription factors (TFs) can work cooperatively with synergistic and antagonist actions as essential mediators of gene expression. Analysis of 148 DE-miRNAs mediated by cocaine and reversely regulated by L-methionine predicted potential significantly differential TFs in the TransmiR v2.0 database. Subsequently, the TRRUST database was used to search the target DEGs to construct transcriptional regulatory networks. Thus, a TF-miRNA-mRNA interaction network was constructed based on the TRRUST and TransmiR analysis and visualized using Cytoscape (Figure 8). The network consisted of 97 nodes and 173 edges. We identified 11 TFs that were significantly regulated by 24 DE-miRNAs. Among them, the TF of Nrf1 targeted by mmu-miR-15b, mmu-miR-486a, mmu-miR-532, mmu-miR-592, mmu-miR-615, and mmu-miR-7054 could regulate the expression of Grin1, which is a hub gene in the addicted associated DEG network interacting with calcium channel genes, glutamate receptor genes, and Egr1 shown in Figure 4C. The TFs of Crebbp targeted by mmu-miR-7054 and Nr3c1 targeted by mmu-miR-15b, mmu-miR-182/3, mmu-miR-195a, mmu-miR-2137, and mmu-miR-7054/8 could regulate Fosb and Nfkbia, the most significantly highly expressed genes affected by cocaine and reversely regulated by L-methionine. Fosb is one of the IEGs following cocaine experience and a transcription factor that regulates reward in the brain (Zhang et al., 2014), suggesting that Fosb may be one of the earliest affected genes by cocaine through transcription factors *via* miRNAs.

### 3.10 Validation of genes reversed by MET

**FIGURE 9:**
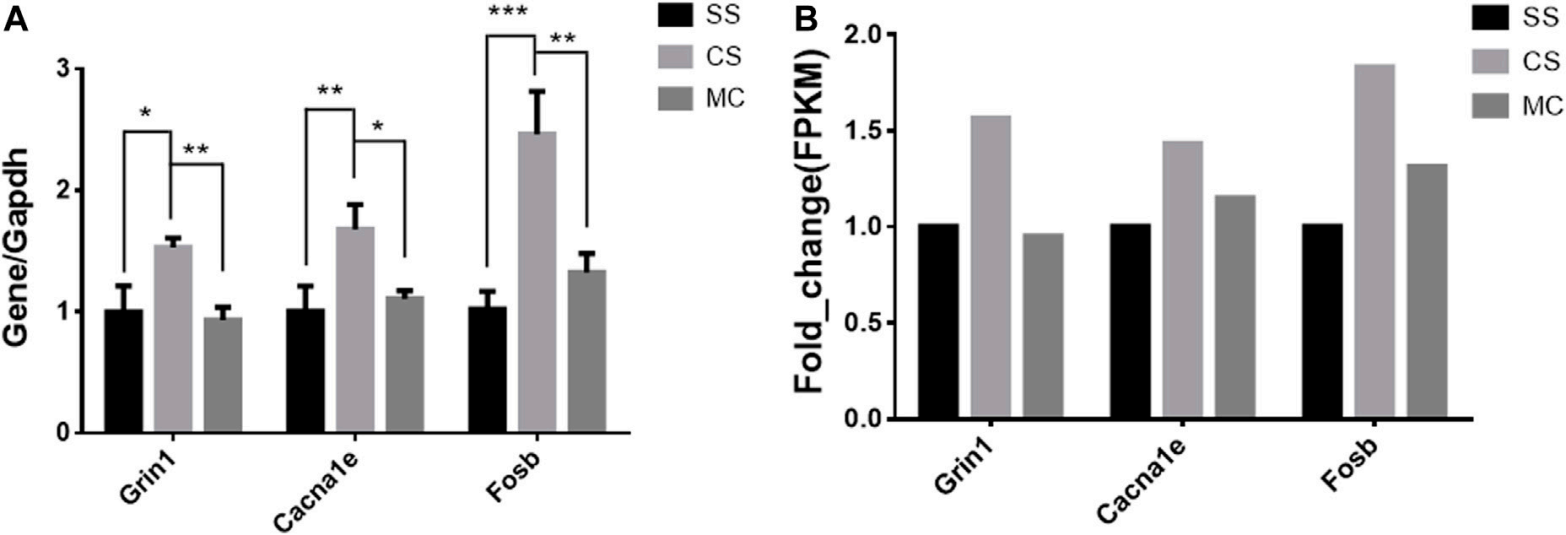
qPCR expression levels of cocaine and MET target genes. **(A)** Normalized mRNA expression quantified by RT-qPCR for genes (Grin1, Cacna1e, and Fosb) whose expression is altered by cocaine treatment (CSvsSS) and reversed by MET (MCvsCS). The results represent the means ± SD of three measurements in each of the two types of experiments; ****p < .0001; ***p < .001; **p < .01; *p < .05. **(B)** Fragments per kilobase of transcript per million mapped reads (FPKM) of Grin1, Cacna1e, and Fosb from mRNA-seq. Note: SS (Saline + Salie), CS (saline + cocaine) and MC (MET + cocaine).

The top-ranked DEGs by fold-change expression were Grin1 (glutamate receptor ionotropic, NMDA 1) and Cacna1e (voltage-dependent R-type calcium channel subunit alpha-1E), which are involved in a variety of calcium-dependent processes including neurotransmitter release, and Fosb (FBJ osteosarcoma oncogene B) play a role in neurogenesis in the hippocampus and in learning and memory-related tasks. An RT-qPCR assay confirmed that the expression of these three genes was significantly altered by cocaine and reversed by MET, consistent with the results of RNA sequencing (Figure 9).

## 4 Discussion

In the current study, we showed that MET inhibited cocaine-CPP by downregulating PFC genes enriched in addiction-related pathways, including the calcium signaling pathway, glutamatergic synapse, the MAPK signaling pathway, the cAMP signaling pathway, and dopaminergic synapse. Long-term exposure to cocaine induces dysfunctional neuroadaptations, which are associated with long-term synaptic potentiation at excitatory synapses throughout the mesolimbic dopamine system, including within the PFC (Go et al., 2016; Joffe and Grueter, 2016; Huang et al., 2017). We showed that the glutamatergic synapse pathway was the top-ranked pathway enriched with DEGs induced by cocaine and was downregulated by L-methionine. It is well demonstrated that gene expression of glutamatergic signaling in the PFC shows different patterns of change according to the regimen of cocaine treatment in cocaine-sensitized mice, mainly in synthesis enzymes and glutamate receptors such as mGluR5, NR1, NR2A, NR2B, NR2C, and glutamate-synthesizing gene kidney-type glutaminase (KGA) (Blanco et al., 2014). Moreover, the genes encoding glutamate receptors change in the hippocampus of humans addicted to cocaine (Enoch et al., 2014). Targeting the glutamatergic system has been suggested as a new pharmacotherapeutic intervention in the treatment of cocaine addiction (White et al., 2016; Ivan Ezquerra-Romano et al., 2018). Thus, our results further demonstrated that MET inhibited cocaine CPP mainly by the glutamatergic synapse system, suggesting its potential therapeutic application in drug addiction.

In addition, the pathways obtained from the PPI network revealed modules labeled as drug addiction-related clusters (M6–M9). Subnetwork-related drug addiction was formed mainly with four subgroups: calcium channel (Cacna1e, Cacna1i, Cacnb1, Cacng4, and Cacng8), glutamate receptor (Grin3a, Grin1, and Grm1), G protein (Gng10, Gng11, and Gng13), and protein kinase C (Prkcb and Prkcg). Combined with previous studies reporting the modulation of Ca^2+^ channels *via* activation of G-protein-coupled receptors and subsequently protein kinase C (PKC) (Zhang et al., 2008; Raifman et al., 2017), we proposed calcium channels and their mediating gene families as a core gene network of cocaine reward. Moreover, calcium channel blockers can prevent or reduce dependence on drugs, particularly their reinforcing actions and withdrawal syndrome (Little, 2021). In this instance, MET has the same function as calcium channel blockers. While the mechanisms related to the calcium channel in drug addiction remain unclear, including changes in the activity of mesolimbic dopamine neurons, genomic effects, and alterations in synaptic plasticity. Future studies including *in vivo* experiments will aim to identify more clues on how the calcium signaling pathway underlies the effects of L-methionine toward cocaine reward.

MicroRNAs could account for the systemic alterations in mRNA and protein expression observed with drug abuse and dependence by repressing mRNA expression through binding to the 3′UTR of their targets. Recently, miRNAs have been suggested to be induced by prolonged cocaine exposure, which drives the escalation of drug use (Most et al., 2014). Importantly, we found that miRNAs target DEGs induced by cocaine and inhibited by MET. These pathways were enriched in GABAergic synapse, glutamatergic synapse, circadian entrainment, axon guidance, and calcium signaling, which were also top-ranked among DEG-enriched pathways, which confirmed that L-methionine inhibited cocaine CPP behaviors by reversing DE-miRNAs and consequently reversing DEGs located in pathways such as the glutamatergic synapses and calcium signaling pathways.

We further analyzed networks comprising the target genes that are regulated by miRNA in the process of L-methionine inhibiting cocaine CPP. Three gene families were identified, including calcium channel (Cacna1c, Cacna1e, Cacna1g, and Cacng8), ephrin receptor (Ephb6 and Epha8), and ryanodine receptor (Ryr1 and Ryr2) genes. Ryanodine receptors form a class of intracellular calcium channels in neurons and mediate the release of calcium ions to regulate numerous neurological processes including synaptic transmission, excitability, learning, and memory (Santulli and Marks, 2015), which further support its core roles in the effects of L-methionine to counteract cocaine.

Our analysis also predicted the presence of Grin1 and Fosb in both the TF-miRNA-mRNA coregulation and PPI networks, with high degrees of interaction. Grin1 reportedly plays a central role in verbal memory and cognitive function and interacts with calcium channels, Egr1, Grin3a, etc. (Ohba et al., 2015). Fosb is one of the IEGs following cocaine experience. Moreover, FosB binds to and regulates target genes by recruiting HDAC and histone methyltransferases, a cofactor for methylation reactions with *S*-adenosyl methionine (SAM) (Nestler, 2008). As the precursor of SAM, whether L-methionine promotes Fosb target genes by recruiting histone methyltransferases requires further investigation.

Comprehensively, we speculate that MET inhibits cocaine-rewarding properties, most likely by upregulating specific miRNAs that inversely regulate their target genes. The genes are mainly involved in pathways for glutamatergic synapses and calcium channels. Our results revealed novel molecular mechanisms of MET that inhibited the rewarding actions of cocaine in brain reward circuitries and provide theoretic support for the development of anti-addiction therapeutics based on MET.

## Data Availability

The datasets presented in this study can be found in online repositories. The names of the repository/repositories and accession number(s) can be found in the article/Supplementary Material.
