# Supplementary material for: Identification of miRNA-mediated gene regulatory networks in L-methionine exposure counteracts cocaine-conditioned place preference in mice
Source: Front Genet. 2023 Jan 19;13:1076156. doi: 10.3389/fgene.2022.1076156 (PMC9893020; doi:10.3389/fgene.2022.1076156)
Supplement: Supplementary file 1 [file Table1.DOCX]

**Supplementary Table S1**. Primer sequences used for qPCR.

| Primers used for QPCR | | |
| --- | --- | --- |
| Gene | Forward (5’-3’) | Reverse (5’-3’) |
| Cacna1e | ATGGACAAGGCTACCACGGAGA | GACTGGCTTCTCCATCCGTCTT |
| Fosb | ACCTGTCTTCGGTGGACTCCTT | TGGCTGGTTGTGATTGCGGTGA |
| Grin1 | CCTTTCAGAGCACACTGTGGCT | CCAGGAAAACCACATGGCAGAG |

**Supplementary Table S2**. Summary of mRNA sequencing raw data.

| category | No. | Total reads | Total mapped reads | Total mapped rate (%) | Unique mapped reads | Unique mapped rate (%) |
| --- | --- | --- | --- | --- | --- | --- |
| MS | #3 | 66099826 | 29006260 | 43.88 | 25518078 | 38.61 |
|  | #4 | 65347112 | 31878334 | 48.78 | 28189726 | 43.14 |
|  | #23 | 65385162 | 36431216 | 55.72 | 32261912 | 49.34 |
| CS | #8 | 67194362 | 35585418 | 52.96 | 31694358 | 47.17 |
|  | #9 | 65563534 | 33643362 | 51.31 | 29932574 | 45.65 |
|  | #10 | 65224228 | 40097792 | 61.48 | 35564308 | 54.53 |
| SS | #12 | 66690546 | 31254348 | 46.86 | 27784038 | 41.66 |
|  | #13 | 65125732 | 30904348 | 47.45 | 27395436 | 42.07 |
|  | #14 | 66491650 | 34213300 | 51.46 | 30353012 | 45.65 |
| MC | #16 | 66038218 | 36531908 | 55.32 | 30975010 | 46.9 |
|  | #17 | 65102418 | 30051190 | 46.16 | 26581794 | 40.83 |
|  | #20 | 65400450 | 32981668 | 50.43 | 29138036 | 44.55 |

**Supplementary Table S3.** List of BP (Biological Process) items of CS (cocaine+saline) group verse SS (saline+saline) with p_value < 0.2(see excel file3).

**Supplementary Table S4.** Top 10 nodes ranked by Betweenness Centrality.

| Name | Betweenness Centrality |
| --- | --- |
| Rps27a | 0.091107 |
| Hras | 0.088338 |
| Cdh1 | 0.064563 |
| Mapt | 0.059382 |
| Acta2 | 0.055342 |
| Grin1 | 0.035561 |
| Isg15 | 0.031983 |
| Smad3 | 0.028277 |
| Nfkbia | 0.0272 |
| Trrap | 0.025454 |

**Supplementary Table S5.** Lists of DEGs in different modules.

| Module | Gene |
| --- | --- |
| M1 | Rps8, Rps24, Rps23, Eef1a1, Rps19, Rpl34, Rps3, Gnb2l1, Naca, Rps7, Rps27a, Rps25, Rps3a1, Rpl27a, Sec61b, Rpl19, Rps12, Rpl37a, Eif3h, Rpl35, Rpl22l1, Rps28, Rplp2, Rps9, Sec61g, Rps17, Rpl17, Rps14, Rps2, Rps21, Rpl36, Rpl27, Rps10, Rpl7a, Eef1a2, Eef1b2, Rpl7, Rpl11, Rpl23, Nsa2, Rpl22, Rpl37, Srp14, Rps27, Rps18, Rps20, Rpl35a, Plec, Rpl9, Rpl26, Nacad, Eif3e, Rps26, Ears2, Tpt1, Rpl13a, Rplp1, Rpl30, Rpl12 |
| M2 | Ndufb6, Cox7b, Ndufb4, Sdhc, Ndufb2, Ndufa4, Cox6c, Usmg5, Sdhd, Atp5l, Uqcrq, Cox8a, Ndufb3, Tomm7, Ndufa7, Ndufb8, Cox6b2, Cox7a2 |
| M3 | Iigp1, Ifi47, Gbp3, Irf7, Ncl, Cxcl10, Gbp7, Ifi44, Dhx9, Ifit1, Gbp6, Larp1, Irgm2, Bst2, Irgm1, Trim30a, Ddx58 |
| M4 | Spp1, Krt19, Hras, Cd34, Cdh2, Cdh1, B2m, Acta2, Lox |
| M5 | Mfap1b, Sf3a2, Rrp12, Ythdc2, Srrm2, Ddx27, Snrpn, Sart1, Lsm7, Dqx1, Sf3b6, Upf1, Prpf6, Rbm19, Bud13, Kri1 |
| M6 | Nr4a3, Egr4, Ube2d3, Chd8, Paip1, Selk, Med31, Med16, Dusp1, Hif3a, Gtf2f1, Havcr2, Med21, Dstn, Actn1, Paf1, Ssrp1, Lamp2, Tnfaip3, Csrp2, Tagln, Lgals3, Commd1, Dcun1d5, Gpx4, Igf1r, Tsc2, Sptan1, Gpx1, Scly, Fosb, Med11, Pdlim2, Fosl2, Ctr9, Trnau1ap, Egr2, Supt5 |
| M7 | Gchfr, Iscu, Cacng4, Pts, Pip4k2b, Cacnb1, Plch2, Qdpr, Inpp5b, Isca2, Cacng8, Pcbd2, Alas2, Gsg1l, Cacna1e, Mtmr4, Cacna1i, Frrs1l, Nfs1, Hspa12a |
| M8 | Eef1e1, Prkcb, Tnnt2, Pkp2, Myh7, Cfl2, Rad51, Gng13, Cenpn, Myh10, Sumo2, Gng11, Ncapg, Prkcg, Gng10, Anln, Spdl1, Ttk |
| M9 | Per2, Ciart, F12, Junb, Dbp, F3, Prcp, Egr1, Serping1, Nr1d1 |

**Supplementary Table S6.** Summary of miRNA sequencing raw data.

| category | No. | Total reads | Clean reads | Percentage (%) |
| --- | --- | --- | --- | --- |
| MS | #3 | 12400744 | 11214089 | 90.43 |
|  | #4 | 11542603 | 10299590 | 89.23 |
|  | #23 | 11952859 | 10633883 | 88.97 |
| CS | #8 | 11556922 | 9272242 | 80.23 |
|  | #9 | 12047119 | 9402614 | 78.05 |
|  | #10 | 11956658 | 9448406 | 79.02 |
| SS | #12 | 11663732 | 8963415 | 76.85 |
|  | #13 | 11600577 | 10288167 | 88.69 |
|  | #14 | 12436566 | 11341914 | 91.2 |
| MC | #16 | 12260115 | 9367763 | 76.41 |
|  | #17 | 11799632 | 9261461 | 78.49 |
|  | #20 | 12287003 | 11320356 | 92.13 |

**Supplementary Table S7.** List of differentially expressed miRNAs in CS, MS and MC groups (see excel file7).
