# Supplementary material for: Identification of miRNA-mediated gene regulatory networks in L-methionine exposure counteracts cocaine-conditioned place preference in mice
Source: Front Genet. 2023 Jan 19;13:1076156. doi: 10.3389/fgene.2022.1076156 (PMC9893020; doi:10.3389/fgene.2022.1076156)
Supplement: Supplementary file 2 [file Image1.pdf]

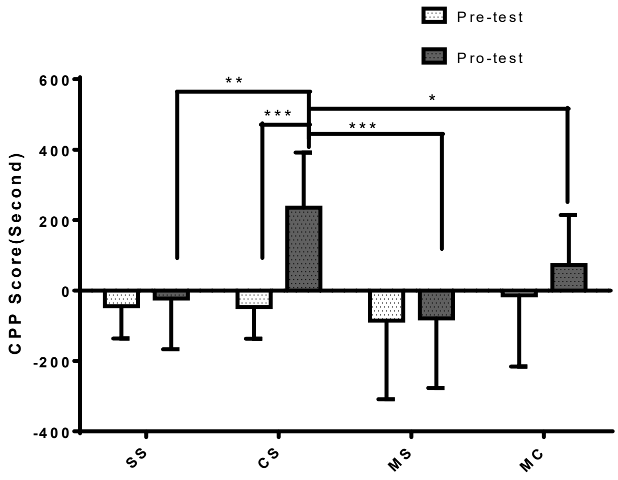

Figure S1. Effect of L-methionine (MET; 200 mg/kg) on behavior in response to cocaine-addicted mice (20 mg/kg, i.p.). CS, saline+cocaine; MC, MET+cocaine; MS, MET+saline; SS, saline+saline. All results represent the mean ± SD of 10 independent determinants (n=10/group) in behavioral test experiments. ***P<.001, **P<0.01, *P<0.05.
